# Benefit finding in men affected by prostate cancer prior to and following radical prostatectomy – a cross-sectional study with a stratified sample

**DOI:** 10.1186/s12885-023-11018-7

**Published:** 2023-06-05

**Authors:** Matthias Jahnen, Patrick Bayer, Valentin H. Meissner, Stefan Schiele, Helga Schulwitz, Jürgen E. Gschwend, Kathleen Herkommer, Andreas Dinkel

**Affiliations:** 1 Department of Urology, Klinikum rechts der Isar, School of Medicine, Technical University of Munich, Ismaninger Str. 22, 81675 Munich, Germany; 2 Department of Psychosomatic Medicine and Psychotherapy, Klinikum rechts der Isar, School of Medicine, Technical University of Munich, Langerstr.3, 81675 Munich, Germany

**Keywords:** Prostate cancer, Benefit finding, Post traumatic growth, Cancer survivors, Survivorship, Radical prostatectomy

## Abstract

**Background:**

Benefit finding (BF) - the occurrence of positive life-changes in the aftermath of traumatic live events - has been repeatedly reported in prostate cancer (PCa) survivors, but it remains unclear in which way BF might vary over time. The current study aimed to investigate the extent of BF and associated factors in different phases of the survivorship continuum.

**Methods:**

In this cross-sectional study, men affected by PCa who were either already treated with radical prostatectomy or going to be treated with radical prostatectomy at a large German PCa center were included. These men were stratified into four groups (prior to surgery, up to 12 months after surgery, 2–5 years and ≥ 6–10 years after surgery). BF was assessed using the German version of the 17-item Benefit Finding Scale (BFS). The items are rated on a five-point Likert scale ranging from 1 to 5. A total mean score ≥ 3 was considered as moderate-to-high BF. Associations with clinical and psychological factors were assessed in men presenting before and in those who participated after surgery. Multiple linear regression was applied to identify intendent determinants of BF.

**Results:**

2,298 men affected by PCa (mean age at survey: 69.5,SD = 8.2; median follow-up: 3 years (25th -75th percentile 0.5-7)) were included. 49.6% of men reported moderate-to-high BF. The mean BF score was 2.91 (SD = 0.92). BF reported by men before surgery did not differ significantly from BF reported by men in the years after surgery (p = 0.56). Higher BF prior to and following radical prostatectomy was associated with higher perceived severity of the disease (pre-surgery: ß = 0.188, p = 0.008; post-surgery: ß = 0.161, p = < 0.0001) and higher cancer-related distress (pre-surgery: ß ? 0.155, p = 0.03; post-surgery: ß = 0.089, p < 0.0001). Post radical prostatectomy BF was also associated with biochemical recurrence during follow-up (ß = 0.089, p = 0.001), and higher quality of life (ß = 0.124, p < 0.001).

**Conclusions:**

Many men affected by PCa perceive BF already soon after diagnosis. The subjective perception of threat or severity associated with the diagnosis of PCa is an essential factor for higher levels of BF, probably more important than objective indicators of the severity of the disease. The early onset of BF and the high degree of similarity of BF reported across the different phases of survivorship suggests that BF is, to a large extent, a dispositional personal characteristic and a cognitive strategy of positively coping with cancer.

## Introduction

Due to an aging population and improvements in early detection, almost 450,000 men are diagnosed with prostate cancer (PCa), requiring subsequent therapy, in Europe annually [1]. Although PCa is most often diagnosed in an early, symptomless stage and long-term survival is common, the diagnosis of PCa and subsequent therapy (e.g. surgery, radiotherapy, or androgen deprivation therapy) have in many cases a profound impact on the life of affected men [2, 3]. These men often perceive a new distrust in their bodies, and therapy side effects such as incontinence, bowel dysfunction, and erectile dysfunction bring about novel challenges for them [4–7]. These adversities have put a growing focus on how to advise men affected by PCa on how to cope with the challenges that occur after the cancer diagnosis and primary therapy. In this regard, research on cancer survivorship has shown that despite the physical and psychological hardships caused by cancer, affected individuals might also report positive life changes when adapting to the disease and its consequences. Such changes might include the strengthening of important relationships or focusing on more meaningful life goals [8]. These positive developments related to diseases and other stressful life events are known as benefit finding (BF) or post-traumatic growth (PTG). There is a high conceptual overlap between BF and PTG [9] and the two terms are often used interchangeably [10, 11]. While there is inconclusive evidence regarding the associations between BF/PTG and sociodemographic characteristics[10, 12], some psychological variables were consistently found to correlate with BF/PTG. A high degree of hope, optimism, meaning-making, self-efficacy, and social support was shown to correlate with high BF, while depressive and anxiety symptoms were associated with lower levels of BF [10, 12]. With regard to disease-related variables, recent systematic reviews reported contradictory findings for cancer type, stage and treatments [10, 12]. This also holds for the association between time since diagnosis and BF/PTG. While the well-known conceptual model of PTG posits that it takes time for perceiving personal growth after a stressful event, most studies investigating BF/PTG after the diagnosis of cancer did not find an effect of time since diagnosis [10, 11, 13]. However, the majority of the studies included in the reviews by Casellas-Grau et al. [10] and Marziliano et al. [11] looked at cancer survivors up to five years after diagnosis and had a sample size below 500 participants. A more recent study comparing men with PCa 5–9 years after diagnosis with men 10–16 years after diagnosis showed significant lower BF in the latter group suggesting that there might be a decline of BF in very long term PCa survivors [14].

In a previous study of our own group [15], we investigated BF in 4252 long-term PCa survivors with a median follow up of 14.8 years after radical prostatectomy, only 9.2% of the participants were less than 10 years after initial treatment. We found that nearly 60% of those long-term PCa survivors reported moderate-to-high BF, Besides the perceived severity of the disease, which emerged as the strongest statistical predictor, younger age at diagnosis was independently associated with BF. As age at diagnosis, age at survey and time since diagnosis were strongly intercorrelated, we decided to drop time since diagnosis from the analyses in this study.

In light of the equivocal results and the limitations of previous studies, we aim to investigate the effect of time since diagnosis more closely in the current study. This study draws on a different sample of PCa survivors than our previous work [15]. It includes short-term and long-term survivors. Furthermore, as there is evidence that BF/PTG occurs even preemptively [16], we also invited PCa patients who were post diagnosis but pre prostatectomy to participate. Further, we aimed at identifying sociodemographic, clinical, and psychological factors independently relating to BF.

## Subjects and methods

### Design and procedure

This is a cross-sectional study that was conducted by surveying men treated for PCa exclusively with radical prostatectomy at the Department of Urology of the Klinikum rechts der Isar, Technical University of Munich between 2009 and 2020. These men were contacted via mail and were asked to complete questionnaires concerning current sociodemographic, clinical, and psychosocial information. Further clinical and pre-surgical information for this analysis was taken from the medical record. All participants provided their written consent to participate in this study. The study was approved by the local ethics committee (Ref. No. 25/20S).

Excluded from the analysis were men who underwent neoadjuvant therapy (n = 82) and men who answered less than 14 items of the BF scale (n = 97). Overall 2298 (prior to surgery n = 221, post-surgery n = 2086) men were included in this analysis.

### Measures

#### Sociodemographic and clinical characteristics

The following sociodemographic data were included in this analysis: age at survey, current partnership, and children. Clinical data included: age at surgery, time since surgery, presence of a second primary cancer, family history of PCa (yes: at least one consanguine relative with PCa vs. no), PSA level at diagnosis, histopathological Gleason grade group, organ-confined stage at RP according to the TNM classification of 2002 (yes: ≤pT3a, N0 and R0 vs. no: (≥ pT3b, N1 and/or R1), biochemical recurrence (PSA level ≥ 0.2 ng/ml) during follow-up, ongoing PCa treatment at survey, American Society of Anesthesiologists Score at surgery and Royal College of Surgeons Charlson comorbidity score at surgery [17].

#### Benefit finding

BF was assessed using the German version of the 17-item benefit finding scale [18, 19]. The scale contains the stem: “Having had prostate cancer…”, followed by 17 different items assessing different potential benefits. Participants were asked to answer the items on a five-point scale ranging from ‘not at all’ [1] to ‘extremely’ [5]. Responses were added up, and divided by the number of answered items in order to calculate the overall mean BF-score, with a higher mean indicating higher benefit finding. A total mean score ≥ 4.0 was considered as “high benefit finding”, and a mean score ≥ 3.0 was considered as “moderate-to-high benefit finding”. The response to the single items was also categorized into high (≥ 4.0) and moderate-to-high (≥ 3) [20].

#### Depression and anxiety

Symptoms of depression and anxiety were assessed using the validated ultra-brief instruments Patient Health Questionnaire-2 (PHQ-2) and Generalized Anxiety Disorder-2 (GAD-2) scale. For both scales (range 0–6), a cut-off score ≥ 3 indicates a clinical level of depression or anxiety, respectively [21, 22].

#### Psychosocial distress

Psychosocial distress was assessed with the short form of the Questionnaire on Distress in Cancer Patients (QSC-R10) using 10 items that capture cancer specific stressors (Book et al., 2011). The response categories range from 0 (does not apply) to 5 (very serious problem). A sum score ≥ 15 indicates clinical distress [23].

### Perceived need for psycho-oncological support

Perceived need for psycho-oncological support was assessed using the single item “Do you currently need psychological support”, with a yes/no response option.

#### Global health status/Quality of life

Quality of life was assessed using two items of the European Organization for Research and Treatment of Cancer Quality of Life Questionnaire (EORTC QLQ-C30). These two items capture the overall health and quality of life in the past week. Participants were asked to answer on a seven-point Likert-scale ranging from `very poor´ [1] to ‘excellent’ [7]. Based on the standardized EORTC formula the mean value of the two items was calculated to a score ranging from 0 to 100. Higher scores indicate a higher quality of life [24].

#### Perceived severity of the disease

The perceived severity of being affected by PCa was assessed with the single item “Having had prostate cancer is one of the worst things that happened to me in my life” (adapted from [25]). Participants were asked to answer on a four-point scale ranging from `strongly disagree´ [1] (no perceived severity) to ‘strongly agree’ [4] (high perceived severity).

### Statistical analysis

Descriptive statistics were calculated for all study variables. In order to investigate the effect of time since diagnosis, we stratified the sample in four groups: before surgery, up to 12 months after surgery, 2–5 years and ≥ 6–10 years after surgery. For all four groups, the average BF score as well as the rate of moderate-to-high and high BF was calculated. Further, item endorsement of the 17 BFS items was assessed in all four groups and tested for significant differences between the groups using chi-square test. Simple linear regression analysis was performed to assess zero-order associations with BF prior to and post-surgery. Additionally, hierarchical multiple linear regression analysis was applied to identify variables independently associated with BF in men post-surgery. Due to the small sample size no multiple linear regression analysis was conducted for men prior to surgery. Statistical significance was set at p < 0.05. All analyses were performed using SAS (Version 9.4).

## Results

2,298 men affected by PCa and primarily treated with radical prostatectomy represent the study sample. Mean age at survey was 69.5 ± 8.2 years. The median time since prostatectomy was 3 years (1st and 3rd quartile: 0.5; 7). 42.5% of men did not have an organ-confined disease stage at surgery and 22.1% of men experienced a biochemical recurrence. Half of the men (50.0%) reported a moderate or high perceived severity of the disease. Every fourth man (25.6%) indicated high cancer-specific distress, while the rate of clinical levels of depressive and anxiety symptoms was much lower (Table 1).

**Table 1:** Sociodemographic, clinical, and psychological characteristics of the study sample (n = 2,298)

	n	%
Sociodemographic factors		
Age at survey (years)M: 69.5 SD: 8.2		
≤ 60	315	13.7
> 60 - ≤70	797	34.7
> 70	1,186	51.6
Partnership		
yes	1,879	91.0
no	186	9.0
Children		
0	358	17.9
≥ 1	1640	82.1
Clinical characteristics		
Age at surgery (years)M: 65.6 SD: 7.7		
≤ 55	221	9.6
> 55 ≤ 65	783	34.1
> 65	1294	56.3
Time since surgery (years)Mdn: 3; 25th -75th Pctl: 0.5-7		
Preoperative	212	9.2
0-≤1	562	24.5
2-≤5	810	35.2
> 5	714	31.1
Second primary cancer		
yes	172	7.5
no	2126	92.5
Family history of PCa		
yes	690	30.0
no	1608	70.0
PSA level at diagnosis (ng/ml) Mdn: 7.3; 25th -75th Pctl: 5.1–10.6		
≤ 4	221	9.6
> 4 ≤ 10	1403	61.1
> 10	672	29.3
ISUP		
≤ 1	314	13.8
2/3	1581	69.5
4/5	381	16.7
Organ-confined stage at RP		
yes	1321	57.5
no	977	42.5
Biochemical recurrence		
yes	496	22.1
no	1745	77.9
Ongoing treatment at survey		
yes	204	9.8
no	1888	90.2
ASA-Score		
I	402	18.5
II	1491	68.4
III	285	13.1
RCS Charlson Score (at surgery)		
0	1509	85.2
1	204	11.5
≥ 2	59	3.2
Psychosocial factors		
Depressive symptoms (PHQ-2)M: 1.0; SD: 1.2		
positive screening (≥ 3)	206	9.1
negative screening (< 3)	2052	90.9
Anxiety symptoms (GAD-2)M: 0.9; SD: 1,1		
positive screening (≥ 3)	169	7.5
negative screening (< 3)	2083	92.5
Distress (QSC-R10) M: 10.0; SD: 8.7		
high ≥ 15	557	25.6
low < 15	1619	74.4
Psychosocial counseling desired		
yes	480	21.5
no	1750	78.5
Quality of life (QLQ-C30)M: 72.5 SD:18.8		
Perceived severity of disease		
no	343	15.2
low	785	34.8
moderate	649	28.8
high	479	21.2

Note: *M*, mean; *SD*, standard deviation; *MED*, median; Pctl, Percentile; *PCa*, prostate cancer; *PSA*, prostate specific antigen; *RP*, radical prostatectomy; *ASA*, American Society of Anesthesiologists; *RCS*, Royal College of Surgeons; *QSC-R10*, Questionnaire on Stress in Cancer Patients; *EORTC QLQ*, European Organization for Research and Treatment of Cancer Quality of Life Questionnaire; *PHQ*, patient health questionnaire; *GAD*, general anxiety disorder.

**Fig. 1:**
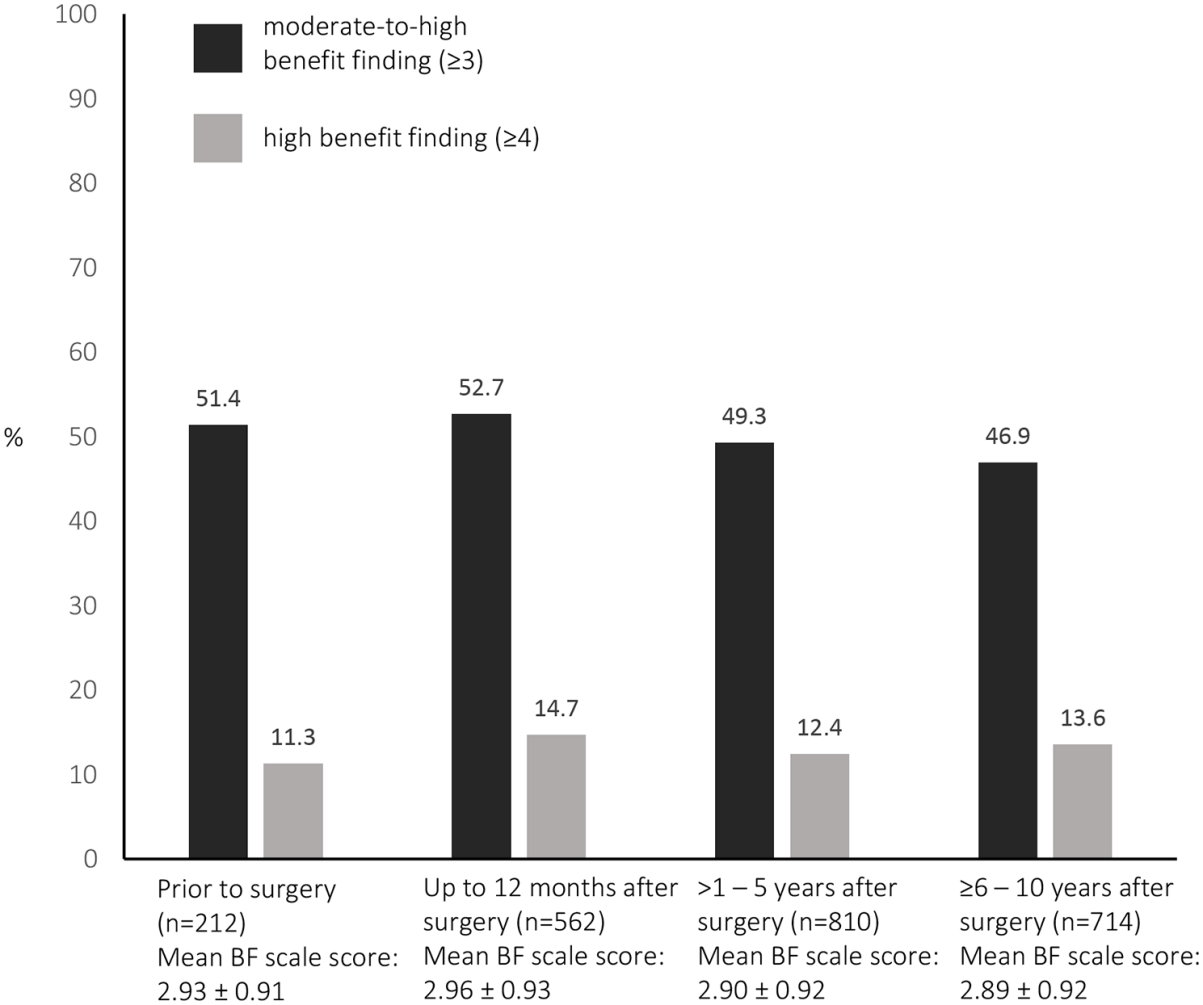
Proportion of men reporting moderate-to-high and high benefit finding in reference to radical prostatectomy (p = 0.56)

**Fig. 2:**
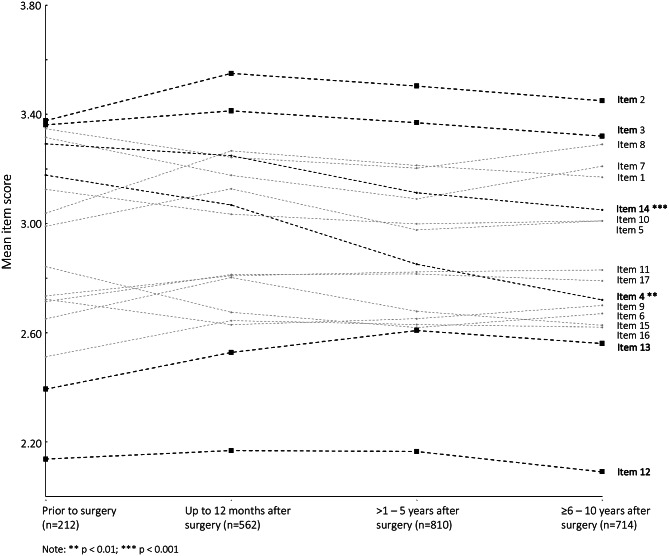
Mean single item score of the benefit finding scale in reference to radical prostatectomy

Overall, 49.6% of men reported moderate-to-high BF and 13.3% reported high BF. The mean BF scale score was 2.91 ± 0.92. With regards to the men surveyed before surgery (n = 212), 51.4% reported moderate-to-high BF and 11.3% high BF. Comparison between men surveyed before surgery and following surgery did not show significant differences in the rates of reported BF (p = 0.56) and no differences in mean BF scale score (prior to surgery: 2.93 ± 0.91; up to 12 months after surgery: 2.96 ± 0.93; >1–5 years after surgery: 2.90 ± 0.92; >5–10 years after surgery: 2.89 ± 0.92) (Figs. 1 and 2).

The most endorsed items across all time periods were “having had prostate cancer has taught me how to adjust to things I cannot change” (mean item score: 3.49 ± 1.11) and ”having had prostate cancer has helped me take things as they come” (mean item score: 3.37 ± 1.15). The least endorsed items across all time periods were “having had prostate cancer has led me to meet people who have become some of my best friends” (mean item score: 2.14 ± 1.18) and “having had prostate cancer has contributed to my overall emotional and spiritual growth” (mean item score: 2.55 ± 1.18). The endorsement of most items was similar in the four groups. However, the items “having had prostate cancer has helped me become more aware of the love and support available from other people” and “having had prostate cancer has brought my family closer together” were endorsed significantly lower by men who were surveyed years after PCa diagnosis and radical prostatectomy (p: < 0.01) (Fig. 2; Table 2).

**Table 2:** Single benefit finding items, mean scores with standard deviation, and frequency of strong endorsement (n = 2261–2293)

Item	Having had prostate cancer…	M	SD	% ≥4
**2**	…**has taught me how to adjust to things I cannot change**	3.49	1.11	59.3
**3**	…**has helped me take things as they come**	3.37	1.15	53.4
8	…has made me realize the importance of planning for my family’s future	3.23	1.31	49.9
1	…has led me to be more accepting of things	3.20	1.11	45.5
7	…has shown me that all people need to be loved	3.17	1.32	46.3
**14**	…**has helped me become more aware of the love and support available from other people**	31.4	1.25	45.2
10	…has taught me to be patient	3.03	1.20	38.6
5	…has made me more sensitive to family issues	3.03	1.20	40.7
**4**	…**has brought my family closer together**	2.90	1.29	36.7
11	…has led me to deal better with stress and problems	2.81	1.17	30.9
17	…has helped me become a stronger person, more able to cope effectively with future life challenges	2.80	1.25	31.7
15	…has helped me realize who my real friends are	2.69	1.36	33.0
9	…has made me more aware and concerned for the future of all human beings	2.67	1.24	29.4
6	…has taught me that everyone has a purpose in life	2.67	1.29	30.1
16	…has helped me become more focused on priorities, with a deeper sense of purpose in life	2.62	1.26	27.6
**13**	…**has contributed to my overall emotional and spiritual growth**	2.55	1.18	24.5
**12**	…**has led me to meet people who have become some of my best friends**	2.14	1.18	16.2

Note: *M*, mean; *SD*, standard deviation; *%≥4* frequency of strong item endorsement.

Simple linear regression analysis revealed an association between higher perceived severity of the disease as well as higher psychosocial distress and higher BF. These associations were significant for men pre surgery (perceived severity of the disease: ß = 0.188, p = 0.008; psychosocial distress: ß = 0.155, p = 0.03) as well as men post-surgery (perceived severity of the disease: ß = 0.161, p < 0.0001; psychosocial distress: ß = 0.089, p < 0.0001) (Table 3).

**Table 3:** Simple regression analysis for benefit finding prior to and following surgery

	Pre-surgery (n = 212)				Post-surgery (n = 2086)			
	**B**	**SE**	**ß**	**p-value**	**B**	**SE**	**ß**	**p-value**
Sociodemographic factors								
Age at survey (years) *(continuous)*	-0.0036	0.0078	-0.0189	0.64	-0.0058	0.0025	-0.0505	0.02
Partnership *(ref. no)*	0.0872	0.3671	0.0249	0.81	0.145	0.0609	0.0536	0.02
Children*(ref. no)*	0.2112	0.1677	0.0881	0.21	0.1237	0.0561	0.0519	0.03
Clinical characteristics								
Second primary cancer*(ref. no)*	-0.2418	0.2841	-0.0586	0.40	-0.0551	0.0755	-0.016	0.47
Family history of PCa*(ref. no)*	-0.0895	0.1282	-0.0481	0.49	-0.0397	0.0445	-0.0196	0.37
RCS Charlson Score (at surgery)*(continuous)*	0.2096	0.1061	0.135	0.05	0.042	0.0379	0.0242	0.27
PSA Lever at diagnosis *(ref. ≤4)*				0.94				0.94
> 4 ≤ 10	0.0069	0.1956	0.0037		-0.0158	0.0709	-0.0083	
> 10	0.0527	0.2129	0.0261		-0.0013	0.0758	-0.0007	
Psychosocial factors								
Perceived severity of disease*(continuous)*	**0.1789**	**0.0664**	**0.1875**	**0.008**	**0.1495**	**0.0202**	**0.1609**	**< 0.0001**
Depressive symptoms (PHQ-2)*(ref. negative screening (< 3))*	-0.1354	0.3074	-0.031	0.66	-0.0067	0.0686	-0.0021	0.92
Anxiety symptoms (GAD-2)*(ref. negative screening (< 3))*	0.2642	0.2427	0.0764	0.28	-0.0512	0.077	-0.0147	0.51
Distress (QSC-R10*(ref. low < 15)*	0.0186	0.0086	0.155	0.03	**0.0093**	**0.0023**	**0.0893**	**< 0.0001**
Psychosocial counseling desired*(ref. no)*	0.0296	0.186	0.0114	0.87	**0.1596**	**0.0489**	**0.0722**	**0.001**
Quality of life (QLQ-C30)*(continuous)*	-0.0387	0.0428	-0.0637	0.37	0.0189	0.0107	0.0389	0.08

Note: *B*, parameter estimate; *SE*, standard error; *ß*, standardized parameter estimate, *ref.*, reference; *PCa*, prostate cancer; *PSA*, prostate specific antigen; *RCS*, Royal College of Surgeons; *PHQ*, patient health questionnaire; *GAD*, general anxiety disorder; highlighted factors indicate statistical significance (p < 0.01).

Hierarchical multiple linear regression analysis revealed an independent association between a higher Charlson comorbidity score at survey and higher BF (ß = 0.052, p = 0.032). Further, biochemical recurrence during follow-up (ß = 0.089, p = 0.001), higher perceived severity of the disease (ß = 0.123, p < 0.0001), and higher cancer-related distress (ß = 0.103, p = 0.005) were independently associated with higher BF. Higher quality of life was also associated with higher BF (ß = 0.124, p < 0.0001). Time since diagnosis was not independently associated with BF. The final model explained 6.1% of the variance (Table 4).

**Table 4:** Multiple hierarchal linear regression analysis for benefit finding following surgery (n = 1541)

	B	SE	ß	p-value	R^2^
STEP 1 Sociodemographic factors					0.006
Age at survey (years) *(continuous)*	-0.006	0.003	-0.053	0.039	
Partnership *(ref. no)*	0.100	0.074	0.035	0.178	
Children*(ref. no)*	0.093	0.061	0.040	0.127	
STEP 2 Presurgical clinical factors					0.012
Age at survey (years) *(continuous)*	-0.007	0.003	-0.061	0.027	
Partnership *(ref. no)*	0.104	0.074	0.036	0.163	
Children*(ref. no)*	0.092	0.061	0.039	0.133	
Time since surgery (years)*(ref. 0-≤1)*					
Preoperative	0.071	0.112	0.017	0.530	
2-≤5	-0.013	0.060	-0.007	0.825	
> 5	-0.008	0.064	-0.004	0.903	
s primary cancer*(ref. no)*	-0.130	0.090	-0.038	0.141	
Family history of PCa*(ref. no)*	-0.060	0.050	-0.031	0.234	
RCS Charlson Score (at surgery)*(continuous)*	0.106	0.045	0.064	0.019	
PSA Lever at diagnosis *(ref. ≤4)*					
> 4 ≤ 10	-0.049	0.080	-0.026	0.538	
> 10	-0.014	0.086	-0.007	0.872	
STEP 3 Postsurgical clinical factors					0.028
Age at survey (years) *(continuous)*	**-0.008**	**0.003**	**-0.071**	**0.009**	
Partnership *(ref. no)*	0.098	0.074	0.033	0.187	
Children*(ref. no)*	0.091	0.061	0.039	0.133	
Time since surgery (years)*(ref. 0-≤1)*					
Preoperative	0.111	0.111	0.027	0.320	
2-≤5	-0.037	0.059	-0.020	0.531	
> 5	-0.034	0.064	-0.018	0.590	
s primary cancer*(ref. no)*	-0.123	0.088	-0.036	0.164	
Family history of PCa*(ref. no)*	-0.050	0.050	-0.023	0.365	
RCS Charlson Score (at surgery)*(continuous)*	0.094	0.045	0.057	0.035	
PSA Lever at diagnosis *(ref. ≤4)*					
> 4 ≤ 10	-0.067	0.079	-0.036	0.400	
> 10	-0.093	0.087	-0.046	0.289	
Biochemical recurrence*(ref. no)*	**0.199**	**0.060**	**0.092**	**0.001**	
Ongoing treatment at survey*(ref. no)*	0.217	0.085	0.070	0.011	
STEP 4 Psychosocial factors					0.061
Age at survey (years) *(continuous)*	-0.005	0.003	-0.045	0.095	
Partnership *(ref. no)*	0.084	0.073	0.029	0.250	
Children*(ref. no)*	0.095	0.060	0.040	0.114	
Time since surgery (years)*(ref. 0-≤1)*					
Preoperative	0.073	0.110	0.018	0.510	
2-≤5	-0.025	0.060	-0.014	0.664	
> 5	-0.027	0.063	-0.015	0.658	
s primary cancer*(ref. no)*	-0.079	0.087	-0.023	0.364	
Family history of PCa*(ref. no)*	-0.045	0.050	-0.023	0.357	
RCS Charlson Score (at surgery)*(continuous)*	0.095	0.044	0.057	0.032	
PSA Lever at diagnosis *(ref. ≤4)*					
> 4 ≤ 10	-0.060	0.078	-0.032	0.442	
> 10	-0.104	0.086	-0.052	0.227	
Biochemical recurrence*(ref. no)*	**0.193**	**0.059**	**0.089**	**0.001**	
Ongoing treatment at survey*(ref. no)*	0.160	0.084	0.052	0.052	
Perceived severity of disease*(continuous)*	**0.113**	**0.025**	**0.123**	**< 0.0001**	
Depressive symptoms (PHQ-2)*(ref. negative screening (< 3))*	-0.070	0.097	-0.022	0.469	
Anxiety symptoms (GAD-2)*(ref. negative screening (< 3))*	-0.153	0.105	-0.045	0.145	
Psychosocial distress (QSC-R10)*(ref. low < 15)*	**0.011**	**0.004**	**0.103**	**0.005**	
Need for psychological support*(ref. no)*	0.106	0.057	0.049	0.063	
Quality of life (QLQ-C30)*(continuous)*	**0.062**	**0.016**	**0.124**	**< 0.0001**	

Note: *B*, parameter estimate; *SE*, standard error; *ß*, standardized parameter estimate, *ref.*, reference; *PCa*, prostate cancer; *PSA*, prostate specific antigen; *RCS*, Royal College of Surgeons; *PHQ*, patient health questionnaire; *GAD*, general anxiety disorder; highlighted factors indicate statistical significance (p < 0.01)

## Discussion

Many prostate cancer survivors are faced with psychological and physical struggles that may lead to fundamental life changes [5]. They transition through different disease phases, characterized by coping with the diagnosis, treatment, recovery, recurrence and further treatment [4, 7]. This suggests that perceiving benefits in surviving cancer might also gradually grow and evolve over the course of a PCa cancer experience. In a previous study of our group on a different sample of German PCa survivors [15], we showed that moderate to high levels of BF can be found in a large proportion of these men (59.7%) up to 15 years after radical prostatectomy. In this previous study, it was argued, that extensive BF can be found many years after PCa diagnosis and primary therapy as a result of a long period of psychological adaptation which might solidify perceived beneficial life changes, suggesting a steady development of BF over time [15]. In the current study, to assess whether differences in BF depend on the time since diagnosis of prostate cancer we stratified our sample into four groups, representing PCa patients who were to undergo initial treatment (close to diagnosis), very short-term survivors (up to 12 months after prostatectomy), short-term survivors (2–5 years after prostatectomy) and long-term survivors (6–10 years).

Results from our current study show that time since diagnosis is not associated with BF. The prevalence of BF was similar across all four groups, contradicting the idea that BF might gradually increase over time. Previous studies that assessed BF in individuals affected by cancer close after initial diagnosis have argued that there might be qualitative differences in BF experienced by asymptomatic individuals who are newly confronted with a cancer diagnosis and BF in those who are living with cancer for many years [8, 26, 27]. While in the first case BF might be a subjective, acute coping mechanism used to deal with a novel stressor, in the latter case BF might actually reflect profound life changes which take time to develop [8]. To further discriminate between BF reported by men newly diagnosed with PCa and BF in men several years after radical prostatectomy, the 17 items of the BF scale were assessed further. Interestingly, comparison across the four groups with regards to single item endorsement revealed that the subject of the benefits reported did not differ much between men who had been newly diagnosed with PCa and men who were diagnosed with PCa and treated with radical prostatectomy years ago. Across all analyzed time periods, items reflecting changes in attitude and mindset towards big life challenges were endorsed the most, while items reflecting spiritual growth and connecting with new people were endorsed least. Significant differences in item endorsement were only found in items reflecting perceived family support as a result of the PCa diagnosis.

To explore whether BF is differently associated with sociodemographic, clinical and psychological characteristics in men asked before and in those surveyed after radical prostatectomy, we applied simple linear regression. Significant associations with BF did not differ substantially between men prior to surgery and men following surgery. In both groups, BF was most strongly associated with cancer-related distress and high perceived severity of the disease, underlining that BF in men closely after diagnosis but prior to therapy and in PCa survivors years after initial therapy seems to be a similar psychological phenomenon. This high level of similarity of BF in men in different phases of the survivorship continuum suggests that BF might be a dispositional personal characteristic, largely independent from external factors. BF seems to be a cognitive coping strategy characterized by reappraisal and goal adaptation.

The theory behind BF and PTG is that individuals affected by critical events experience sudden life changes which might also impact their life in a meaningful and positive way despite the stress of adversity [8, 13, 28]. However, a substantial burden is necessary in order to trigger a perceived deviation from one’s life path which might be then accompanied by the experience of BF [8, 11, 15, 29]. Multiple regression analysis emphasized the necessity for such sufficient stressors as it showed higher cancer-related distress and higher perceived severity of the disease to be independently associated with higher BF. The perceived severity of the disease is a highly subjective assessment of one’s personal cancer experience which integrates the subjective appraisal of the current illness with one’s previous life experiences. This further suggests that BF is not only determined by the weight of current adversity but also by individual characteristics and previous life experiences. Cancer-related distress was assessed using the QSC-R10 questionnaire which focuses on current physical and psychological symptoms, lack of support as well as fear of the future disease course. Men newly diagnosed with non-metastasized prostate cancer who are treated with radical prostatectomy experience rarely any specific cancer-related symptoms [5]. However, the psychological burden of the diagnosis, side effects from therapy and the fear of disease progression and recurrence are for some men a significant strain on their (mental) health [30, 31]. Men who experience their PCa disease in such a way seem to contemplate more about their health situation and might therefore also show an increased awareness of BF.

Multiple linear regression analysis also showed an association between BF and biochemical tumor recurrence measured by a new PSA increase during follow up. The detection of an increasing PSA is proof of a biochemical tumor recurrence and makes often a demanding follow-up therapy necessary. For many men, PCa recurrence is often more psychologically demanding than initial diagnosis as it makes further adjustments in life necessary and might therefore trigger changes in cancer-related self-perception as well as perceived BF [7, 32].

Results of this analysis have to be considered within certain limitations. Our cross-sectional data allows the comparison of different men to different points of their cancer experience. However, the individual BF trajectory of men affected by PCa, who pass through different disease and treatment phases, needs to be further assessed in longitudinal studies. Further, although a broad set of clinical and psychological variables were investigated, our final model explained only 6% of the variance. This suggests that BF might only depend marginally on situational clinical and psychological factors and more on dispositional variables that were not included in the current study.

Due to the cross-sectional design, causal assumptions on the development and effects of BF after radical prostatectomy cannot be drawn and it remains unclear whether potential unknown moderators effect certain variables. Moreover, by only investigating men affected by PCa who were primarily treated with radical prostatectomy generalization towards men that received different treatment options such as radiation therapy is limited and implications for other cancer types must be treated with caution.

## Conclusions

Many men affected by PCa perceive benefits as a result of their cancer experience already soon after initial diagnosis as well as in the years following treatment with radical prostatectomy. Our results support the view that the subjective perception of threat or severity associated with the diagnosis of prostate cancer is an essential factor for higher levels of BF, probably more important than objective indicators of the severity of the disease. BF already occurs early after the diagnosis of prostate cancer, suggesting that BF is to a large extent a dispositional personal characteristic and a cognitive strategy of positively coping with cancer. Clinicians might consider exploring BF with patients who report being affected by their disease to strengthen the psychological adaptation in all phases of PCa survivorship.

## Data Availability

All available data are included into the manuscript.
